# A Rare Case of Chronic Invasive Pulmonary Aspergillosis

**DOI:** 10.7759/cureus.49944

**Published:** 2023-12-05

**Authors:** Vikram B Vikhe, Devansh Khandol, Aniket A Garud

**Affiliations:** 1 Department of General Medicine, Dr. D. Y. Patil Medical College, Hospital and Research Centre, Pune, IND; 2 Department of Pharmacology, Rasiklal M. Dhariwal Institute of Pharmaceutical Education and Research, Pune, IND

**Keywords:** idiopathic portal cavernoma, acute on chronic pancreatitis, type 2 diabetic mellitus with diabetic neuropathy, young male, chronic invasive pulmonary aspergillosis

## Abstract

Invasive pulmonary aspergillosis (IPA) and invasive aspergillosis (IA) are two examples of the broad clinical spectrum of *Aspergillus *infection. It mainly affects severely immunocompromised hosts, while immunocompetent people can sometimes be affected, especially those receiving treatment in the intensive care unit (ICU) for emergency cases with few instances of chronic cases. The risk factors in ICU patients for aspergillosis include intubated patients receiving hot and humidified air, viral infections like covid, and influenza, and diseases like diabetes, chronic obstructive pulmonary disease, etc. A case of 35-year-old male reported to us with a complaint of stomach discomfort that was acute and non-progressive in the epigastric area, radiating to the back, not accompanied by fever, and not linked with loose stools/vomiting. In addition, the patient experienced a nonproductive cough for two days that was not associated with dyspnea or chest discomfort. He had a high-resolution computed tomography (HRCT) thorax, which revealed a single pulmonary nodule in the left lung's middle zone; histology of the same nodule biopsy material revealed that it was caused by *Aspergillus*. He had an abdominal ultrasound, which revealed portal vein thrombosis, dilated periportal tortuous veins, evident peri splenic and mesenteric collaterals, and significant splenomegaly - suggestive of portal cavernoma formation with chronic liver parenchymal disease. Our patient has a past history of alcohol use disorder for the last 15 years due to which the patient has had recurrent episodes of acute pancreatitis for the last three years which has now progressed to chronic pancreatitis, also the patient has been diabetic for the last 10 years on insulin for the same. A patient with multiple comorbidities, such as cirrhotic portal cavernoma, type 2 diabetes, diabetic neuropathy, and acute and chronic pancreatitis, is the subject of our case study on chronic IPA.

## Introduction

*Aspergillus *infection has a wide clinical range, including invasive pulmonary aspergillosis (IPA) and its disseminated extrapulmonary form, invasive aspergillosis (IA). It primarily affects highly immunocompromised hosts, although it can also impact immunocompetent individuals, particularly those with acute disorders being treated in the intensive care unit (ICU) and, less frequently, [1] those with chronic diseases. However, post-mortem investigations have shown that *Aspergillus *infections are among the most often missed diagnoses [2,3], not just in hematologic patients, but also in those with chronic obstructive pulmonary disease (COPD), liver cirrhosis, or on long-term steroids [4].

## Case presentation

A man, 35 years old, came to us with severe epigastric pain for three days which was acute progressive, radiating to the back, associated with fever, not associated with loose stools or vomiting. The patient also had a cough without expectoration which was present for two days, acute, progressive, not associated with breathlessness, chest pain, and no blood in the sputum**.** The patient had no history of rash, sore throat, cold, headache, or burning micturition. The patient has alcohol use disorder for 15 years with a daily consumption of 150g of ethanol. For 10 years patient has been diabetic on insulin 10 IU subcutaneously three times a day, he also has a known case of recurrent acute on chronic pancreatitis for the last three years attributed to chronic alcohol consumption for which the patient has been admitted to the hospital four times in the last two years. On clinical examination, the patient was afebrile, with normal vitals, saturation, and blood sugar levels. No other abnormal signs were seen on general examination. On per-abdomen examination, the abdomen was soft/non-tender on palpation with no guarding/rigidity, and no signs of ascites or dilated veins were seen. The spleen was palpable 2cm below the costal margin. There was no hepatomegaly. On cardiovascular system examination, normal heart sounds were heard. No murmur was heard. On respiratory system examination, there was reduced air entry in the left infraclavicular region. Normal breath sounds were heard over the rest of the lung fields bilaterally.

**Figure 1:**
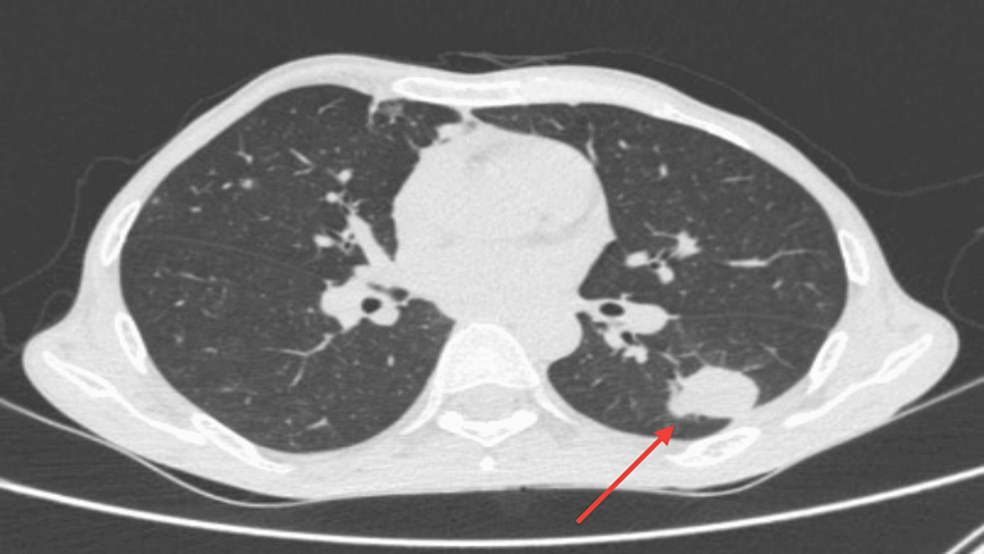
Single ill-defined pulmonary nodule (seen with red arrow) with an irregular spiculated margin showing subtle peripheral post-contrast enhancement with subpleural thickening seen in adjacent pleura in the left lower lobe in the superior segment as seen in this axial CT thorax section.

**Figure 2:**
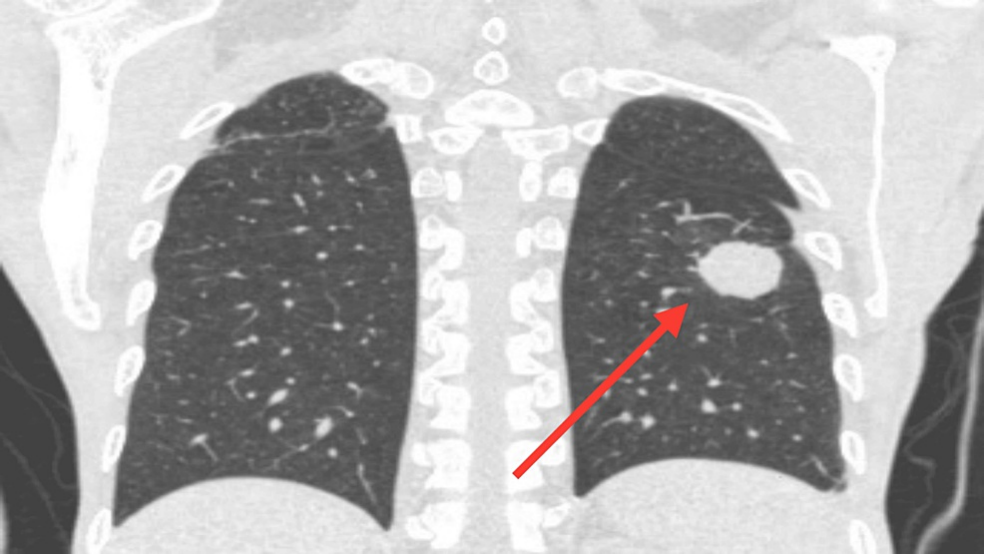
Solitary pulmonary nodule in the left lower lobe in the superior segment as seen in the current coronal section of CT thorax figure with red arrow.

Chest X-ray was suggestive of round homogenous opacity in the left middle zone. On the laboratory parameters, the hemogram along with serum electrolytes, liver, and renal functions was normal. His blood sugar level was 218mg/dl. Serum amylase and lipase were 230 U/L and 990 U/L respectively. CRP and ESR were elevated. Sputum and bronchoalveolar lavage (BAL) fluid studies were done. There were no acid-fast bacilli or fungal hyphae on routine microscopy, the culture showed no growth of any organism and the cartridge-based nucleic acid amplification test (CBNAAT) was negative for pulmonary tuberculosis. Contrast-enhanced computed tomography (CECT) thorax was suggestive of a fairly ill-defined pulmonary nodule in the superior segment of the left lower lobe which was solitary with an irregular spiculated margin. It showed subtle peripheral post-contrast enhancement with subpleural thickening which was seen in adjacent pleura. Neoplastic or fungal aspergilloma aetiology needs consideration as shown in Figure 1 (axial section) and Figure 2 (coronal section). There were no features suggestive of old healed pulmonary tuberculosis or new active pulmonary tuberculosis.  ​​​​​

**Figure 3:**
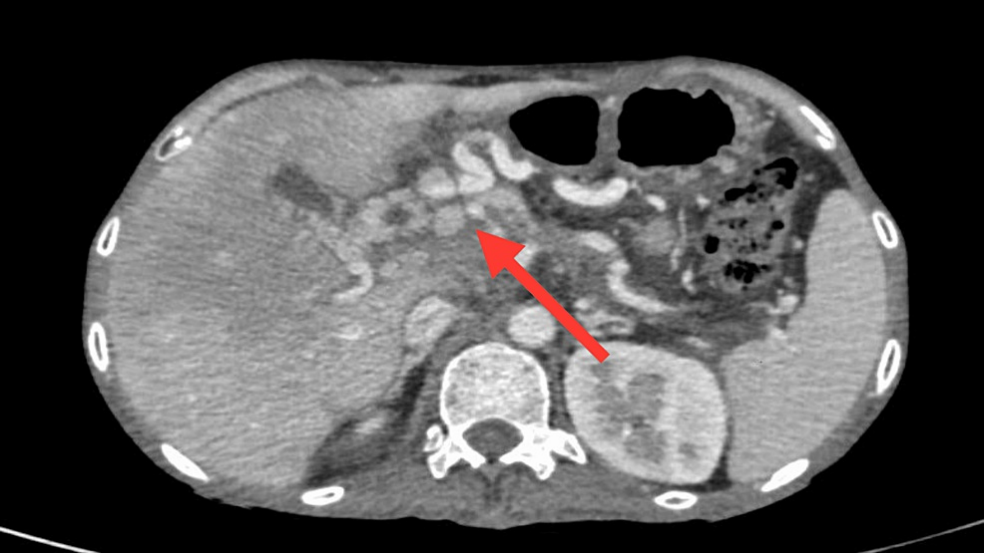
This CECT abdomen axial section shows the portal vein which is completely replaced by a tuft of dilated tortuous collaterals - cavernoma formation as shown by the red arrow. CECT: contrast-enhanced computed tomography

**Figure 4:**
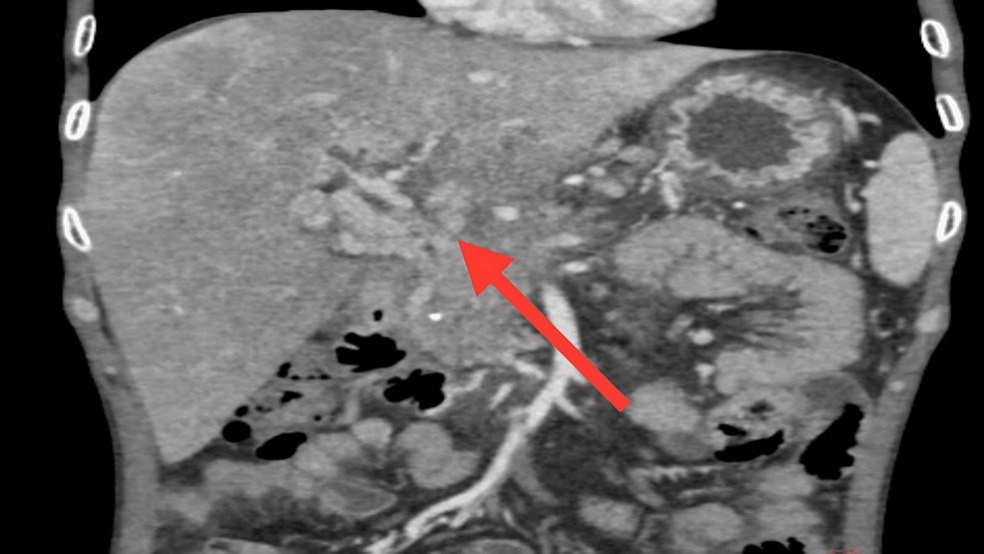
This coronal CECT abdomen section shows coarse echotexture of the liver with portal cavernoma formation (as seen with red arrow) with multiple dilated tortuous collaterals in the peri-pancreatic region, peri-gastric region, para oesophageal region, and at splenic hilum. CECT: contrast-enhanced computed tomography

Ultrasonography of the abdomen was suggestive of a liver shrunken in size with coarse echotexture with a tortuous portal vein. It is associated with multiple periportal, mesenteric, and peri-splenic collaterals. There is gall bladder oedema. There is mild splenomegaly. CECT abdomen and pelvis were suggestive of acute or chronic pancreatitis, with few small pseudocysts. The portal vein is completely replaced by a tuft of dilated tortuous collaterals known as cavernoma formation. There is also hepatomegaly with transient hepatic attenuation differences due to portal hypoperfusion. There is mild splenomegaly. Multiple dilated tortuous collaterals are seen in the peri-pancreatic region, peri-gastric region, para-esophageal region, and at splenic hilum as seen in Figure 3 (axial section) and Figure 4 (coronal section).

On repeat chest X-ray, the patient’s nodule was not improving despite antimicrobial therapy of cefepime and tazobactam combination 1.125g intravenously two times a day for seven days leading to suspicion of malignancy/fungal infection - so for the confirmation of the same CT-guided lung biopsy was taken and sent for histopathological examination.

**Figure 5:**
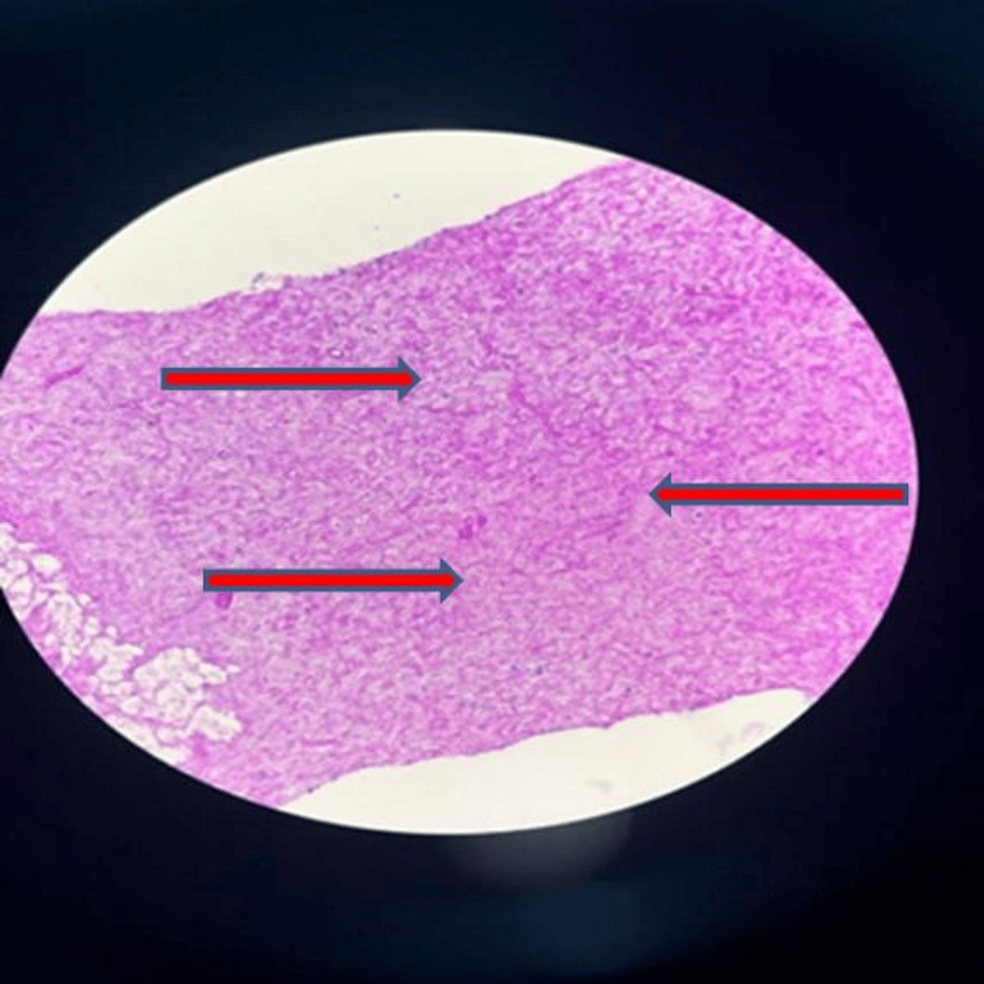
Multiple slender fungal hyphae with acute angle branching amidst the background of the necrotic area.

**Figure 6:**
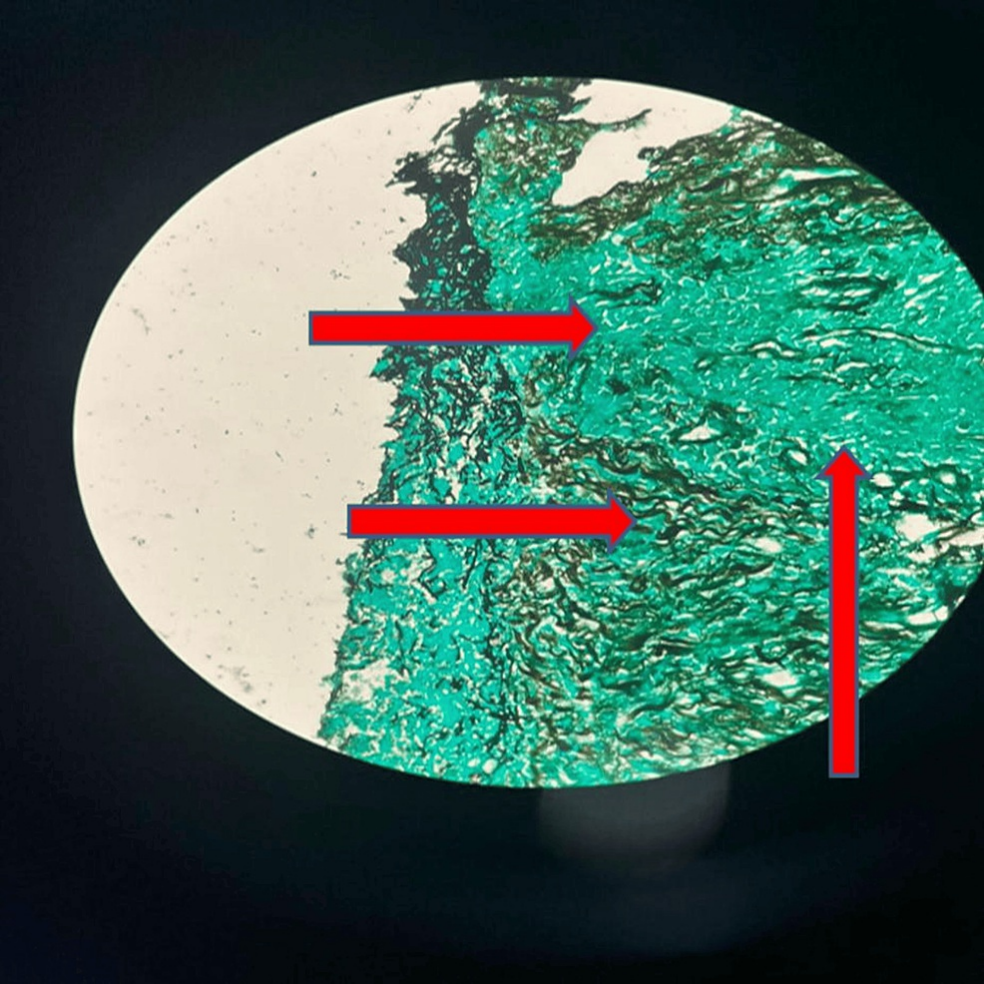
On Gomori methenamine stain (GMS) of the same tissue section, it showed brown-coloured fungal hyphae with acute angle branching.

Histopathological examination of lung biopsy showed multiple fragmented cores showing predominantly areas of necrosis with focal adjacent fibro collagenous tissue. The tissue in Figure 5 shows infiltration by neutrophils, lymphocytes, and a few plasma cells. The necrotic area reveals multiple slender fungal hyphae showing acute angle branching (shown with red arrows).** **Figure 6 shows a tissue section with Gomori methenamine stain (GMS), showing (with multiple arrows) brown-coloured fungal hyphae with acute angle branching.

The patient started treatment with an injection of voriconazole 400mg intravenous stat dose followed by 200mg intravenous two times a day for 14 days and then was switched to an oral tablet form of 200mg two times a day for seven more weeks. Repeat CECT thorax after six weeks showed minimal resolution in the pulmonary nodule thus necessitating continuation of treatment for at least four more months as the patient is immunocompromised due to multiple comorbidities requiring long-term therapy for clinical and radiological resolution. Our patient did not have typical radiological features of IPA plus sputum and BAL fluid both were negative for aspergillosis making the suspicion less likely and diagnosis delayed as occurs in most cases with aspergillosis resulting in delayed resolution and increased morbidity and mortality.

## Discussion

**Figure 7:**
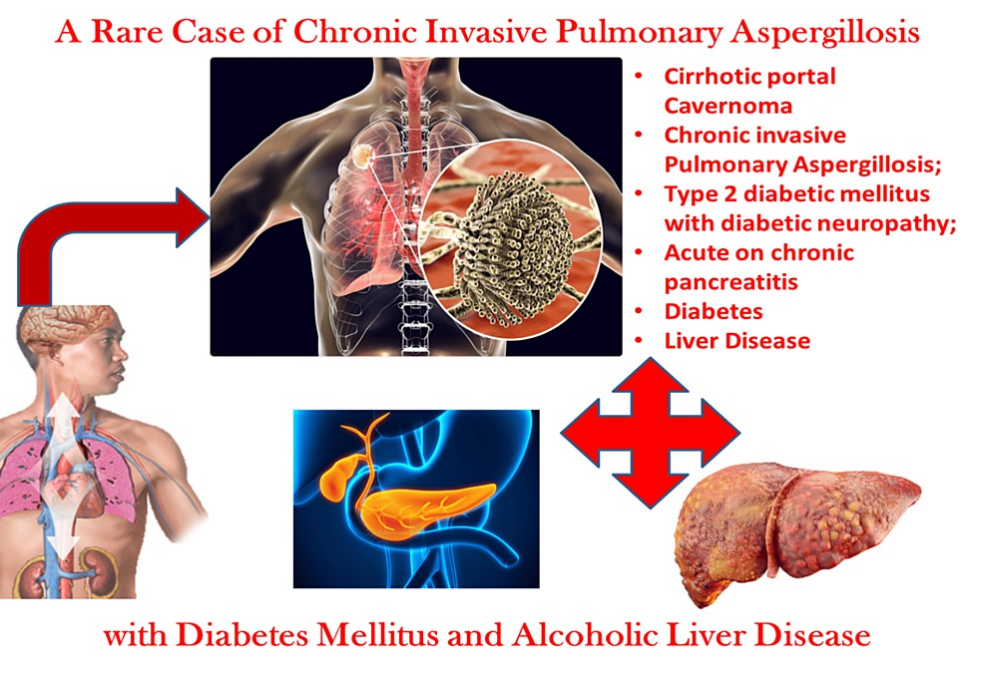
Graphical animated representation of all the comorbidities the patient has which increased the chances of getting aspergillosis which is an opportunistic infection. Image credits: Dr. Aniket Garud

Patients with chronic pancreatitis, acute or severe liver sickness, or type 2 diabetes are often unrecognized as a high-risk group for IA, leading to delays in diagnosis. Accordingly, the majority of cases - including our patient - are initially mislabelled as having pneumonia caused by bacteria, delaying the identification and management of IA. This observation highlights the challenges of establishing the clinical diagnosis in patients who are not neutropenic. The pathophysiology of immune-related illness (IA) varies significantly between patients with and without neutropenia. Although angio-invasive lesions are widespread and have a high fungal burden in neutropenic patients, the pathogenesis in individuals who are not neutropenic and are treated with steroids is caused by a detrimental inflammatory host response, resulting in a reduced fungal load in the pulmonary parenchyma and, less frequently, infection dissemination [5]. *Aspergillus *species cultures obtained from respiratory secretions are not very sensitive in diagnosing invasive infections. Furthermore, lung CT scans are not as useful in individuals who are not neutropenic since they do not show any typical abnormalities such as cavitation, the air crescent sign, or the halo sign. Additionally, *Aspergillus *infections can arise in cases of severe respiratory distress syndrome or over an existing case of atelectasis [4]. Patients with cirrhosis may experience further complications as a result of their immune system being considerably compromised by corticosteroid medication, allogeneic blood product transfusions, poor glycemic control, dialysis, etc. The following graphical animated representation is seen in Figure 7 which shows a rare culmination of all comorbidities from portal cavernoma to uncontrolled diabetes to acute or chronic pancreatitis all of which are individual risk factors for aspergillosis (the possibility of all three in a single patient is extremely rare).

Galactomannan (GM) detection is a recent modality for the diagnosis of IA [6]. With a sensitivity of 88% and a threshold index of 0.5, BAL GM was found to be particularly helpful in identifying pulmonary aspergillosis [7] in patients in critical care units. According to all of these findings, GM detection in the BAL fluid may be a helpful diagnostic procedure for patients without neutropenia who exhibit pneumonia symptoms but do not improve after receiving initial broad-spectrum antibiotic treatment. Early therapy implementation lowers mortality and slows the course of the illness. Now, voriconazole is the preferred medication; nevertheless, while being a CYP450 inhibitor, it has several pharmacological [8] interactions and may be hepatotoxic. Nonetheless, among individuals who are not neutropenic, its use is linked to a lower death rate. One benefit of isavuconazole is that it has fewer interactions with cytochrome P450 (CYP). Other alternatives include echinocandin, posaconazole and itraconazole. A minimum of six to twelve weeks of treatment is advised by the Infectious Diseases Society of America (IDSA) [9]. Patients with IPA who have an aspergilloma, chronic hemoptysis non-responders to medical management and are considered low-surgical risk patients should be operated on. Mortality rates have been decreasing in the last few years. Every three to six months, follow-up should be done to assess the quality of life, clinical and radiological evolution, and immunoglobulin G (IgG) levels against *Aspergillus fumigatus*, which can drop but never reach normal levels [10] again.

## Conclusions

The rates of morbidity and mortality are high in IPA and IA. While immunosuppressed patients are the primary victims, immunocompetent people are also believed to be vulnerable. The lack of distinct clinical symptoms makes diagnosis difficult, and immunocompetent people in particular need to be treated with extreme caution to detect IPA early and prevent its progression to IA. To enable the diagnosis and timely treatment initiation, a diagnostic algorithm is necessary. IA is typically a potentially fatal side effect of diabetes mellitus, chronic pancreatitis, or serious liver disease. Medical professionals should be able to identify this potentially fatal side effect in patients with these specific diseases, provide the right antifungal therapy, and lower the associated death rate - which, despite advancements, remains unacceptably high in this patient population.
